# Holly (*Ilex latifolia* Thunb.) Polyphenols Extracts Alleviate Hepatic Damage by Regulating Ferroptosis Following Diquat Challenge in a Piglet Model

**DOI:** 10.3389/fnut.2020.604328

**Published:** 2020-12-15

**Authors:** Pengwei He, Hongwei Hua, Wei Tian, Huiling Zhu, Yulan Liu, Xiao Xu

**Affiliations:** Hubei Key Laboratory of Animal Nutrition and Feed Science, Wuhan Polytechnic University, Wuhan, China

**Keywords:** holly polyphenols extracts, liver, diquat, oxidative stress, ferroptosis, weaned piglets

## Abstract

**Background:** Holly (*Ilex latifolia* Thunb.) polyphenols extracts (HPE) contain high amounts of polyphenols, including phenolic acids, triterpenoids, tannic acids, and so on, which have strong antioxidant function. This experiment was aimed to explore the protective effect and mechanism of HPE against hepatic injury induced by diquat.

**Methods:** Thirty-two weaned piglets were allotted by a 2 × 2 factorial experiment design with diet type (basal diet vs. HPE diet) and diquat challenge (saline vs. diquat). On the 21st day, piglets were injected with diquat or saline. One week later, blood samples were collected. Then all piglets were slaughtered and hepatic samples were collected.

**Results:** Dietary HPE supplementation improves hepatic morphology, the activities of plasma aspartate aminotransferase, alanine aminotransferase, and glutamyl transpeptidase, and enhances hepatic anti-oxidative capacity, while it regulates the expression of ferroptosis mediators (transferrin receptor protein 1, heat shock protein beta 1, solute carrier family 7 member 11, and glutathione peroxidase 4) in diquat-challenged piglets.

**Conclusion:** These results indicate that dietary HPE supplementation enhances hepatic morphology and function, which is involved in modulating antioxidant capacity and ferroptosis.

## Introduction

In recent years, metabolic syndrome has become a significant public health tissue. Liver, as the largest solid organ in our body, has many important functions, such as detoxification process, multiple synthesis, and secretions of endogenous compounds (1, 2). After absorption from the intestine, the liver is the first organ involved in metabolism of dietary nutrients. An abundant amount of reactive oxygen species (ROS) is produced in the liver, and hence the liver is likely to be affected by oxidative stress (3). ROS can induce the animals under oxidative stress, which is implicated in various types of hepatic diseases.

Excessive ROS consume the endogenous antioxidants, which subsequently cannot counteract the over-produced ROS (4). ROS interact with all cellular macromolecules, especially the polyunsaturated fatty acids, which are the main composition of membrane structure, and can be easily attached by ROS through lipid peroxidation leading to cell death (5).

Although cell death is vital for fundamental physiological processes, it is frequently induced in varies stresses and diseases (6). Ferroptosis is the latest identified form of cell death in 2012, which is induced by loss of the lipid repair enzyme glutathione peroxidase 4 (GPX4) activity and then accumulation of lipid-based ROS (7). This type of cell death has its genetical, biochemical, and morphological characteristics, which is different from other cell death types, such as apoptosis, unregulated necrosis, and necroptosis (7). Increasing evidence shows that lipid peroxidation plays a critical role in mediating ferroptosis. Therefore, the relative molecules and signals in lipid peroxidation participate in ferroptosis regulation (8). However, the current studies in ferroptosis are focused in human cancer and neuropathy (9, 10). There are few reports about ferroptosis in the liver under oxidative stress status.

Polyphenols in plants are a kind of secondary metabolite that can provide protection against invasive pathogens and ultraviolet-induced damage (11). Some research has reported that polyphenols exert many beneficial effects on health in humans and rodent models (12, 13). The polyphenols include more than 8,000 different compounds with the common structure, namely the phenol ring (14). The polyphenols can be separated to the flavonoid-type and the non-flavonoid-type polyphenols according to the number of phenol rings and the structural elements that bind these rings to one another (11). *Ilex latifolia* Thunb. is called Da Ye Dong Qing in Chinese and widely consumed in China and other Southeast Asia countries (15). *I. latifolia* Thunb. became popular due to its public acceptance as highly advantageous nutrition and many beverage functions, such as an antioxidant, an anti-obesity agent, and so on (16). In recent years, some studies have shown that the extracts from *I. latifolia* Thunb. contain high amounts of polyphenols, including phenolic acids, triterpenoids, tannic acids, and so on (17). A large number of studies have shown that polyphenol-rich plant extracts or isolated polyphenolic compounds relieved experimentally induced oxidative stress (18, 19). Polyphenols can directly clear ROS as well as increase the activities of various antioxidant enzymes (20).

To explore whether holly polyphenols extracts (HPE) could alleviate hepatic injury by regulating anti-oxidative capacity and ferroptosis, the piglets were fed a basal diet with or without HPE, followed by intraperitoneal administration of diquat to trigger liver oxidative stress and damage in this study (21). We reveal that HPE supplementation improves hepatic morphology, enhances hepatic anti-oxidative capacity, and reduces the expression of ferroptosis mediators in diquat-challenged piglets.

## Materials and Methods

### Animal and Experimental Design

An animal trial was conducted in agreement with the Animal Scientific Procedures Act 1986 (Home Office Code of Practice. HMSO: London January 1997) and the EU regulation (Directive 2010/63/EU). Experimental procedures were approved by the Animal Care and Use Committee of Wuhan Polytechnic University (Wuhan, China). A total of 32 weaned crossbred barrows [Duroc × Landrace × Large White, 35 ± 1 d, 8.16 ± 0.68 kg initial body weight (BW)] were used in the current study. Pigs were solely housed in stainless steel metabolic cages (1.80 × 1.10 m^2^) with free access to food and water in an environmentally controlled house. The ambient temperature was maintained at 22–25°C. The corn-soybean meal basal diet was accepted by the National Research Council requirements (22). The basal diet was also supplemented without or with 250 mg/kg of a commercial holly (*I. latifolia* Thunb.) polyphenols extracts product containing 65.5% total polyphenols, and the main ingredients were phenolic acids and tannins.

The trial was conducted as a 2 × 2 factorial design. Pigs were fed a basal or HPE diet for 21 days, and then they were intraperitoneal injection of diquat (dibromide monohydrate, Chem Service, West Chester, PA) at 100 mg/kg BW in saline or the same volume of saline. The two factors were diet type (basal or HPE diet) and oxidative stress (diquat or saline), respectively.

### Sample Collection

Seven days after injection of diquat or saline solution, blood samples were collected from the jugular vein and placed into heparinized vacuum tubes. Then the blood samples were centrifuged to harvest plasma. Plasma samples were stored at −80°C for analysis of biochemical parameters. After blood collection, all pigs were slaughtered with sodium pentobarbital (80 mg/kg BW), and the liver samples were collected instantly. One piece of liver sample was put in fresh 4% paraformaldehyde/phosphate buffered saline for 24 h to analyze histology. The rest was frozen in liquid nitrogen and transferred to a −80°C freezer for further analysis.

### Plasma Biochemical Indicators

The activities of plasma aspartate aminotransferase (AST), alanine aminotransferase (ALT), and glutamyl transpeptidase (GGT) were determined according to the previous method (20).

### Liver Histology

The liver pieces were dehydrated, embedded, and stained with hematoxylin and eosin after a 24-h fixation. The hepatic injury was measured according to Chen et al. (23).

### ATP, ADP, and AMP Concentrations in the Liver

The adenosine triphosphate (ATP), adenosine diphosphate (ADP), and adenosine monophosphate (AMP) concentrations in the liver of piglets were detected with high performance liquid chromatography (HPLC) in accordance with our previous study (24). Total adenine nucleotide (TAN) and adenylate energy charges (AEC) were calculated by the following equations: TAN = ATP + ADP + AMP; AEC = (ATP + 0.5 ADP)/(ATP + ADP + AMP).

### Anti-Oxidative Capacity of the Liver

Total antioxidative capacity (TAOC), malondialdehyde (MDA), activities of glutathione peroxidases (GSH-PX), and reductive glutathione (GSH) of liver samples were determined using commercial kits from Nanjing Jiancheng Bioengineering Co. The antioxidative capacity was measured according to Chen et al. (23).

### Transmission Electron Microscope (TEM) Observation of the Liver

Moderate liver samples were soaked in 0.1 mol/L phosphate buffer solution and stored in a 4°C refrigerator. Then, the livers of the piglets were dissected and fixed in 2.5% glutaraldehyde at 4°C for over 2 h, after which the samples were washed three times with phosphate buffer solution (pH 7.3). Following, the samples were fixed with 1% osmium tetroxide about 2 h, rewashed three times with phosphate buffer solution and dehydrated with graded concentrations of ethanol (50, 70, 80, 90, and 100%). After soaking with a 1:1 mixture of acetone and resin for 1 h, the samples were soaked with a 1:2 mixture of acetone and resined for 2 h, then embedded with pure resin and sliced. Staining with uranyl acetate and lead acetate, the slices were observed and photographed with a HT7700 TEM (Hitachi Co. Ltd., Japan).

### mRNA Expression Analysis by Real-Time PCR

The methods for total RNA isolation, quantification, reverse transcription, as well as real-time PCR were according to Xu et al. (25). The primer pairs for amplification of target genes are presented in Table 1. The expression of the target genes relative to housekeeping gene (glyceraldehyde-3-phosphate dehydrogenase; GAPDH) was analyzed by the 2^ΔΔ*CT*^ method. Relative mRNA abundance of each target gene was normalized to the piglets fed basic diet and injected with 0.9% NaCl solution.

**Table 1:** Primer sequences used for real-time PCR.

**Gene**	**Forward (5^**′**^-3^**′**^)**	**Reverse (5^**′**^-3^**′**^)**	**Annealing temperature (**°**C)**	**Product length (bp)**	**Accession numbers**
*TFR1*	CGAAGTGGCTGGTCATCT	TGTCTCTTGTCTCTACATTCCT	60	231	NM_214001.1
*HSPB1*	CTCGGAGATCCAGCAGACT	TCGTGCTTGCCCGTGAT	60	120	NM_001007518
*SLC7A11*	GCCTTGTCCTATGCTGAGTTG	GTTCCAGAATGTAGCGTCCAA	60	178	XM_021101587.1
*GPX4*	CTGTTCCGCCTGCTGAA	ACCTCCGTCTTGCCTCAT	60	218	NM_214407.1
*GAPDH*	CGTCCCTGAGACACGATGGT	GCCTTGACTGTGCCGTGGAAT	60	194	AF_017079.1

*TFR1, transferrin receptor protein 1; HSPB1, heat shock protein beta 1; SLC7A11, solute carrier family 7 member 11; GPX4, glutathione peroxidase 4; GAPDH, glyceraldehyde-3-phosphate dehydrogenase*.

### Protein Abundance Analysis by Western Blot

The methods for protein abundance measurement in the liver were referred to Xu et al. (25). Briefly, the liver samples were homogenized in lysis buffer and centrifuged to collect the supernatants for Western blot and protein assay. Hepatic proteins were separated on a polyacrylamide gel and transferred onto polyvinylidene difluoride membranes. The membranes were blocked for non-specific binding for at least 60 min with 3% BSA in TBS/Tween-20 buffer, washed and incubated overnight (12–16 h) at 4°C with primary antibodies. Specific primary antibodies included rabbit anti-transferrin receptor protein 1 (TFR1, 1:1000; 86 kDa, #70R-50471; Fitzgerald, Rd. Sudbury, Acton, MA, USA), goat anti-solute carrier family 7 member 11 (SLC7A11, 1:1000; 55 kDa, #ab60171; Abcam, Cambridge, MA, USA), rabbit anti-glutathione peroxidase 4 (GPX4, 1:1000; 20 kDa, #10005258; Cayman Chemical Company, Rd. Ellsworth, Ann Arbor, MI, USA), and mouse anti-β-actin antibody (1:1000, 43 kDa, #A2228; Sigma-Aldrich, St. Louis, MO, USA). The membranes were then incubated with anti-rabbit/anti-mouse/anti-goat IgG HRP-conjugated secondary antibody for 120 min at room temperature (21–25°C). The relative protein abundance of target proteins (TFR1, SLC7A11, GPX4) were expressed as the ratio of target protein/β-actin protein.

### Statistical Analyses

All data were analyzed as a 2 × 2 factorial design by ANOVA using the general linear model procedures (GLM) of SAS (SAS Inst. Inc., Cary, NC). The statistical model included the effects of diquat challenge (saline or diquat) and diet (basal or HPE), as well as their interactions. Data were presented as means and SEMs. When there was a significant or trend interaction between the two effects, *post-hoc* testing was conducted using Duncan's multiple comparison tests. Differences were considered to be significant if *P* < 0.05. Instances in which 0.05 < *P* ≤ 0.10 were considered as trends.

## Results

### Hepatic Morphology Observation

**Figure 1:**
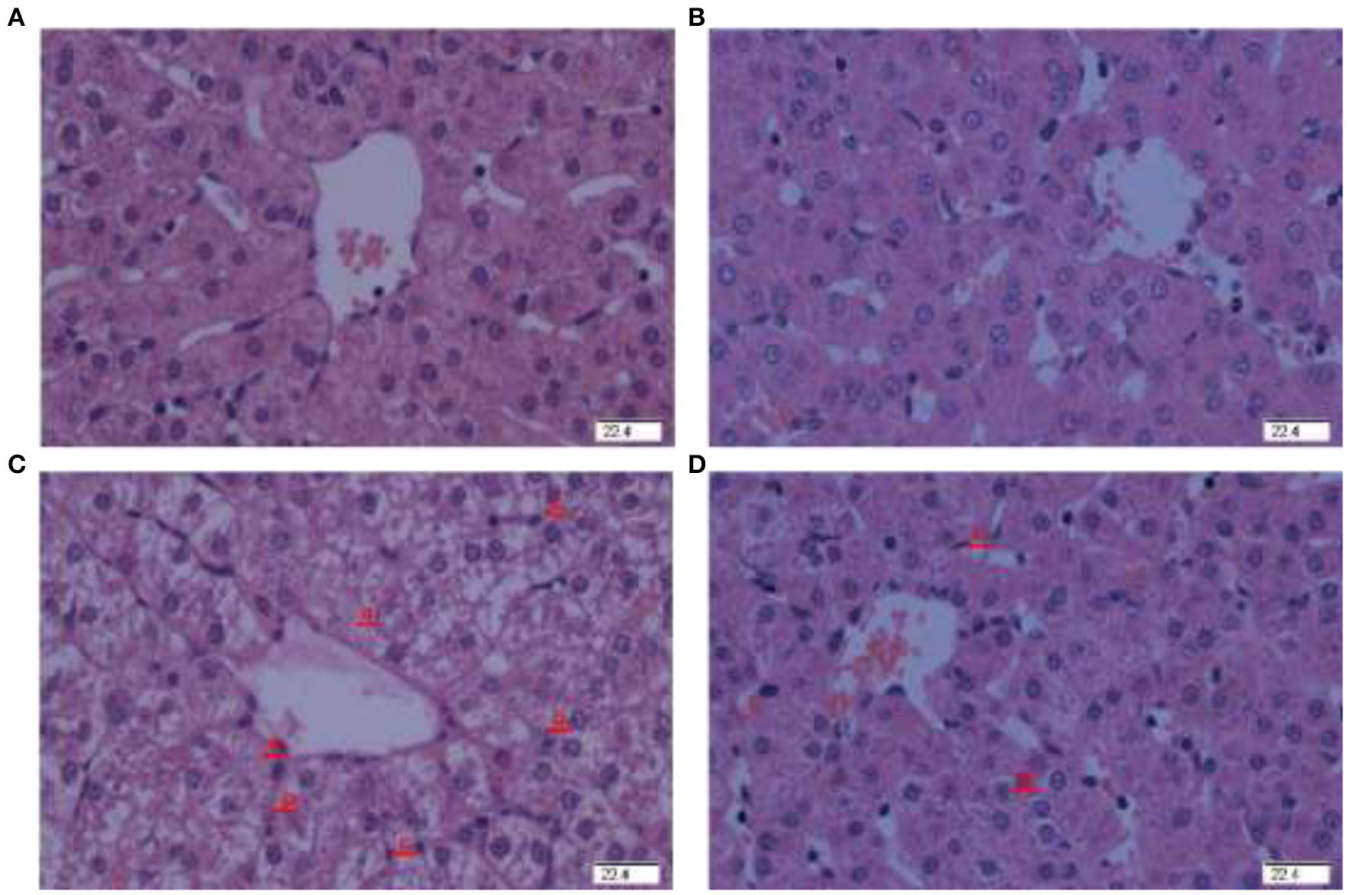
Effect of holly polyphenols extracts (HPE) supplementation on hepatic morphology after 7 d diquat challenge in weanling[-2mm]Q7 piglets. Representative liver photomicrographs are shown. **(A)** Pigs fed a basal diet and treated with 0.9% NaCl solution. **(B)** Pigs fed a HPE diet and treated with 0.9% NaCl solution. No obvious pathological changes were observed. **(C)** Pigs fed the same basal diet and challenged with diquat. Significant pathological changes of liver damage such as hepatic spindle cells disappear (a), hepatocyte caryolysis (b), hepatocyte karyopycnosis (c), hepatic cell cords arrangement in disorder were found. **(D)** Pigs fed a HPE diet and challenged with diquat. Liver damage was significantly alleviated. *n* = 8 (1 pig/pen). Original magnifications 400×. Scale bars = 22.4 μm.

There was no obvious pathological change in the piglets injected with saline and fed basal (Figure 1A) or HPE supplementation diet (Figure 1B). However, pathological changes of liver damage such as hepatocyte caryolysis, karyopycnosis, hepatic spindle cells disappear and hepatic cell cords arrangement in disorder were found in the piglets injected by diquat and fed basal diet (Figure 1C). Compared to the piglets injected with diquat and fed basal diet, liver damage was relieved in the piglets injected with diquat and fed HPE supplementation diet (Figure 1D).

### Plasma Biochemical Parameters

There was a significant interaction between diquat challenge and HPE supplementation for AST, ALT, and GGT activities (*P* < 0.05, Table 2). Supplementation with HPE significantly decreased AST, ALT, and GGT activities in the piglets challenged with diquat (*P* < 0.05).

**Table 2:** The plasma biochemical parameters of the piglets fed holly polyphenols extracts (HPE) diet with diquat challenge.

**Item**	**Saline**		**Diquat**		**SEM**	***P*****-value**		
	**Control**	**HPE**	**Control**	**HPE**		**HPE**	**Diquat**	**Interaction**
AST (U/L)	40.3^b^	37.8^b^	64.7^a^	41.7^b^	6.6	0.162	0.172	0.002
ALT (U/L)	60.4^b^	52.4^b^	74.2^a^	59.7^b^	6.0	0.217	0.121	0.013
GGT (U/L)	39.6^b^	30.5^c^	58.3^a^	31.1^c^	4.8	0.031	0.205	0.021

*N = 8 (1 pig/pen)*.

*SEM, standard error of mean*.

a−c *Labeled means in a row without a common letter differ, P < 0.05. ALT, alanine aminotransferase; AST, aspartate aminotransferase; GGT, glutamyl transpeptidase*.

### Hepatic ATP, ADP, and AMP Content

There was a significant interaction between diquat challenge and HPE supplementation for the hepatic ATP concentration, AEC, and AMP/ATP ratio (*P* < 0.05, Table 3). Supplementation with HPE significantly increased hepatic ATP concentration and AEC in the piglets challenged with diquat (*P* < 0.05). However, the AMP/ATP ratio in the piglets fed HPE was significantly reduced compared with the piglets fed basal diet and challenged with diquat (*P* < 0.05).

**Table 3:** The liver ATP, AMP, and ADP contents (μg/g wet wt) of the piglets fed holly polyphenols extracts (HPE) diet with diquat challenge.

**Item**	**Saline**		**Diquat**		**SEM**	***P*****-value**		
	**Control**	**HPE**	**Control**	**HPE**		**HPE**	**Diquat**	**Interaction**
ATP	597^a^	592^a^	536^b^	612^a^	22	0.146	0.465	0.002
ADP	121	117	128	114	10	0.245	0.836	0.643
AMP	126	124	132	112	14	0.422	0.654	0.879
TAN	844	833	796	838	29	0.239	0.358	0.125
AEC	0.779^b^	0.781^b^	0.754^c^	0.798^a^	0.008	0.035	0.203	0.003
AMP/ATP	0.211^b^	0.209^b^	0.246^a^	0.183^b^	0.015	0.005	0.685	0.001

*N = 8 (1 pig/pen)*.

*SEM, standard error of mean*.

a−c *Labeled means in a row without a common letter differ, P < 0.05. ATP, adenosine triphosphate; ADP, adenosine diphosphate; AMP, adenosine monophosphate; TAN = ATP + ADP + AMP; AEC = (ATP + 0.5 ADP)/(ATP + ADP + AMP)*.

### Hepatic Antioxidant Capacity

There was a significant interaction between diquat challenge and HPE supplementation for hepatic TAOC and reduced GSH amount (*P* < 0.05, Table 4). Supplementation with HPE significantly enhanced hepatic TAOC and increased reduced GSH amount in the piglets challenged with diquat (*P* < 0.05). Moreover, diquat challenged significantly increased hepatic MDA amount and supplementation with HPE significantly reduced hepatic MDA amount in the piglets (*P* < 0.05).

**Table 4:** The liver antioxidative capacity of the piglets fed holly polyphenols extracts (HPE) diet with diquat challenge.

**Item**	**Saline**		**Diquat**		**SEM**	***P*****-value**		
	**Control**	**HPE**	**Control**	**HPE**		**HPE**	**Diquat**	**Interaction**
TAOC	3.13^a^	3.17^a^	2.54^b^	3.16^a^	0.26	0.031	0.247	0.006
GSH-PX (U/mgprot)	211	202	200	215	28	0.774	0.897	0.254
GSH (mgGSH/gprot)	49.5^a^^,^ ^b^	52.2^a^^,^ ^b^	36.4^b^	58.6^a^	8.2	0.040	0.287	0.001
MDA (nmol/mgprot)	3.01	2.40	5.27	3.07	0.70	0.015	0.007	0.152

*N = 8 (1 pig/pen)*.

*SEM, standard error of mean*.

a, b *Labeled means in a row without a common letter differ, P < 0.05. TAOC, total antioxidative capacity; GSH-PX, glutathione peroxidases; GSH, reduced glutathione; MDA, malondialdehyde*.

### Hepatocytes Transmission Electron Microscope Observation

**Figure 2:**
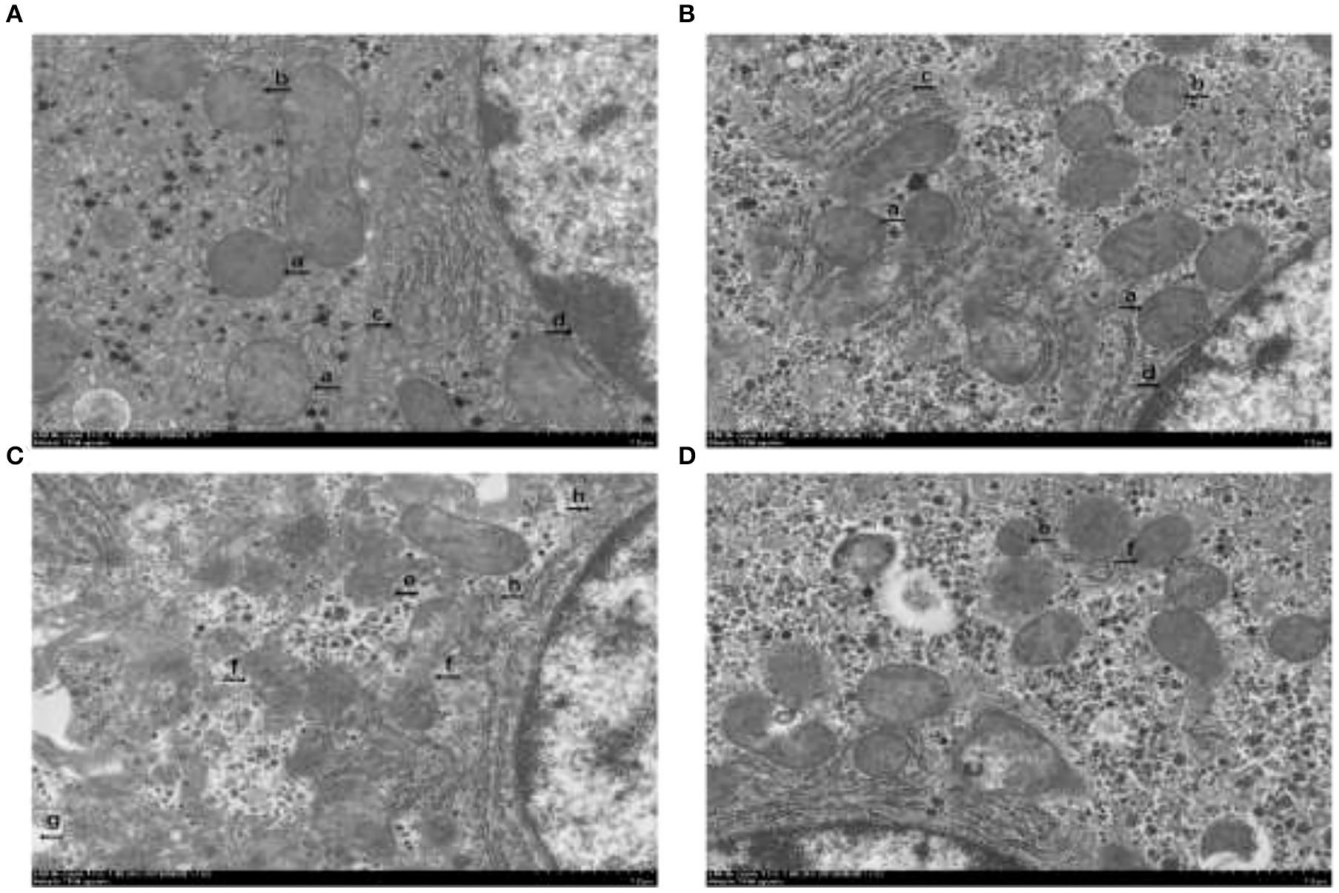
The effect of dietary holly polyphenols extracts (HPE) supplementation on hepatocyte ultrastructure after 7 d diquat challenge in weanling pigs. Representative hepatocyte ultrastructures are shown. **(A)** Pigs fed a basal diet and treated with 0.9% NaCl solution. **(B)** Pigs fed a HPE diet and treated with 0.9% NaCl solution. **(A)** and **(B)** were no obvious ferroptosis characteristics. Presented as complete mitochondria (a), mitochondria with distinct cristae (b), normal rough endoplasmic reticulum (c), karyotheca integrity (d). **(C)** Pigs fed the same basal diet and challenged with diquat. Significant ferroptosis characteristics were observed, such as mitochondrial pyknosis (e), mitochondrial outer membrane rupture (f), mitochondrial cristae reduction (g), and slightly dilatations of rough endoplasmic reticulum (h) were found. **(D)** Pigs fed a HPE diet and challenged with diquat.

There were no obvious characteristics of hepatocytes ferroptosis in the piglets without the diquat challenge (Figures 2A,B). Nevertheless, characteristics of ferroptosis, such as mitochondrial pyknosis, mitochondrial cristae reduction, rough endoplasmic reticulum dilatations, and karyotheca deformation separated from cytoplasm, were found in the piglets challenged with diquat and fed basal diet (Figure 2C). Compared with the piglets challenged with diquat and fed basal diet, hepatocytes ferroptosis was attenuated in the piglets challenged with diquat and fed HPE diet (Figure 2D).

### Hepatic mRNA Expression of Key Genes Related to Ferroptosis

There was a significant interaction between diquat challenge and HPE supplementation for hepatic transferrin receptor protein (*TFR1*), heat shock protein beta 1 (*HSPB1*), solute carrier family 7 member 11 (*SLC7A11*), and glutathione peroxidase 4 (*GPX4*) mRNA expression (*P* < 0.05, Table 5). Supplementation with HPE significantly reduced hepatic *TFR1* mRNA expression in the piglets challenged with diquat (*P* < 0.05). Meanwhile, supplementation with HPE significantly increased hepatic *HSPB1, SLC7A11*, and *GPX4* mRNA expression in the piglets challenged with diquat (*P* < 0.05).

**Table 5:** The liver mRNA expression of ferroptosis-related signals of the piglets fed holly polyphenols extracts (HPE) diet with diquat challenge.

**Item**	**Saline**		**Diquat**		**SEM**	***P*****-value**		
	**Control**	**HPE**	**Control**	**HPE**		**HPE**	**Diquat**	**Interaction**
*TFR1*	1.00^b^	1.10^b^	1.68^a^	1.14^b^	0.17	0.135	0.021	0.013
*HSPB1*	1.00^b^	1.03^b^	0.99^b^	2.14^a^	0.11	0.002	0.014	<0.001
*SLC7A11*	1.00^b^	0.88^b^	1.07^b^	1.48^a^	0.17	0.616	0.365	<0.001
*GPX4*	1.00^b^	0.95^b^	1.12^b^	3.82^a^	0.31	0.035	0.022	<0.001

*N = 8 (1 pig/pen)*.

*SEM, standard error of mean*.

a, b *Labeled means in a row without a common letter differ, P < 0.05. TFR1, transferrin receptor protein 1; HSPB1, heat shock protein beta 1; SLC7A11, solute carrier family 7 member 11; GPX4, glutathione peroxidase 4*.

### Hepatic Protein Abundance Related to Ferroptosis

**Figure 3:**
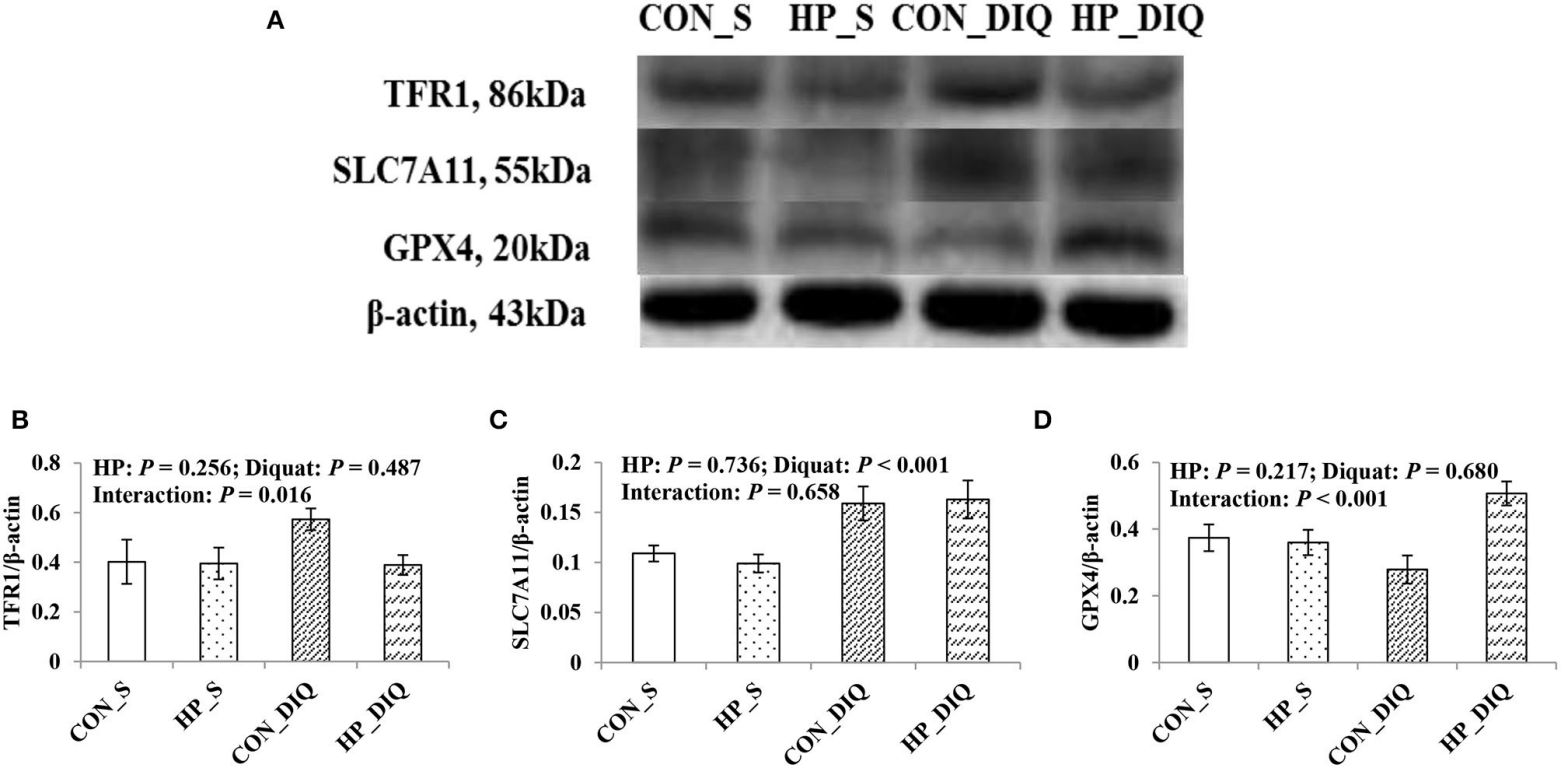
The effect of dietary holly polyphenols extracts (HPE) on protein expression of TFR1 **(B)**, SLC7A11 **(C)**, and GPX4 **(D**) of weaned pigs after 7 d diquat challenge. The bands were the representative Western blot images **(A)**. Values are mean and pooled SEM, *n* = 8 (1 pig/pen). CON_S, piglets fed the basal diet and injected with saline; HP_S, piglets fed HPE diet and injected with saline; CON_DIQ, piglets fed the basal diet and challenged with diquat; HP_DIQ, piglets fed HPE diet and challenged with diquat.

There was a significant interaction between diquat challenge and HPE supplementation for hepatic TFR1 and GPX4 protein abundance (Figure 3, Supplementary Files). Supplementation with HPE significantly reduced hepatic TFR1 abundance, while it increased GPX4 abundance in the piglets challenged with diquat (*P* < 0.05). Moreover, the piglets challenged with diquat had significant hepatic SLC7A11 abundance compared with the piglets without challenge (*P* < 0.05).

## Discussion

This experiment was aimed to explore whether HPE could alleviate diquat-induced hepatic injury in a weanling piglet model and to explore the regulation mechanism of ferroptosis in hepatocytes death. Dietary supplementation with HPE improved hepatic morphology and function enhanced the status of energy metabolism and anti-oxidant capacity in the liver. Moreover, dietary supplementation with HPE relieved the extent of hepatocytes ferroptosis by regulating the expression of gene and protein associated with ferroptosis.

Hepatic morphological observation and plasma biochemical analysis (ALT, AST, and GGT activities) are direct methods to evaluate hepatic dysfunction (23). In the present study, pathological liver with hepatocyte caryolysis, karyopycnosis, and altered hepatic cord arrangement was observed in the piglets challenged with diquat and fed a basal diet. However, only mild hepatic injury was observed in the piglets challenged with diquat and fed a HPE supplemented diet. Similar results also occurred in the plasma biochemical parameters. The liver is the primary metabolic organ and also considered an organ with immune function in human and animals, in which altered indices in the liver affect many body's function. The negative change of hepatic indices demonstrated that diquat treatment successfully induced oxidative stress in the piglets. Dietary HPE supplementation attenuated diquat-induced hepatic pathological morphology and dysfunction. Some previous studies also showed a protective effect of HPE on ALT and AST activities in plasma and hepatic morphology (26, 27).

ATP is the most direct source of energy in the body. It can participate in a variety of cellular functions and meet the energy requirements during the early response to a stress (28). Some previous studies showed that HPE improved lipid metabolism (29). HPE reduced intracellular triglyceride and cholesterol contents in rats (30). Feng et al. also reported that HPE inhibited liver weight and deposition of lipid (27). In the present study, the increased ATP content may be caused by decomposition of lipid by HPE to produce more energy to satisfy hepatic function and energy requirement.

Diquat can induce oxidative stress and decrease antioxidant capacity in the piglets (21). TAOC reflects the body or organ cumulative effect of all antioxidants (31). MDA is an important marker for reflecting the degree of lipid peroxidation and the extent of cellular damage (32). GSH is a tripeptide with a gamma peptide linkage between the carboxyl group of the glutamate side chain and cysteine, and the carboxyl group of the cysteine residue is attached by normal peptide linkage to glycine (33). Meanwhile, GSH is regarded as a cofactor of GSH-PX, involved in neutralizing H_2_O_2_ and lipid hydroperoxides, and the alterations in the GSH redox state responds to the oxidative stress and redox status of the body (33). The current results indicated that HPE increased hepatic TAOC, reductive GSH content, and reduced MDA content in the piglets challenged with diquat. Many previous researches have reported that HPE had strong antioxidant capacity (13, 16). In agreement with our study, Zhao et al. demonstrated that polyphenols in Kuding tea increased the amount of reductive GSH and decreased the amount of MDA in a HCl/ethanol-induced gastric injury mice model (34). In our study, a diquat challenge induced an oxidative stress, which led to a lipid metabolism disorder and disrupted the oxidative state homeostasis. Supplementation with HPE strongly prevented the hepatic injury *via* its antioxidant function.

The classical programmed cell death, including apoptosis, autophagy, and programmed necrosis, is referred to be death of a cell in any pathological format when mediated *via* an intracellular program (35). In 2012, Dixon et al. reported an original and non-apoptotic cell death forms from rat brain slices, which relates to the oxidative stress process closely, called ferroptosis, and is morphologically, biochemically, and genetically distinct from apoptosis, necrosis, and autophagy (7). Ferroptosis is a programmed cell death form dependent on iron, as well as originated by the malfunction of the glutathione-dependent antioxidant defenses, resulting in unchecked lipid peroxidation and eventual cell death (36). The representative features of cell ferroptosis include lipid peroxidation, mitochondrial pyknosis, mitochondrial outer membrane rupture, and mitochondrial cristae reduction (7). The transmission electron microscope observation in our study clearly showed characteristics of ferroptosis in hepatocytes, such as mitochondrial pyknosis, mitochondrial outer membrane rupture, mitochondrial cristae reduction, rough endoplasmic reticulum dilatations, and karyotheca deformation separated from cytoplasm in the piglets challenged with diquat, which illustrated diquat-induced hepatic ferroptosis. Moreover, dietary HPE supplementation alleviated hepatic ferroptosis in the organelle level of the piglets.

Ferroptosis is activated by several intracellular and extracellular factors. TFR1, also known as CD71, is a receptor protein encoded by the transferrin receptor (TFRC) gene (37). When ferroptosis occurs, the protein can be used as a carrier to transfer ferric iron (Fe^3+^) into the inner cell membrane. HSPB1 is a chaperone of the small heat shock protein (sHsp) group among ubiquitin, α-crystallin, Hsp20, and others. The main functions of sHsps include chaperone activity, thermotolerance, inhibition of apoptosis, regulation of cell development, and differentiation. HSPB1 can reduce the concentration of Fe^3+^ by inhibiting the express of TFR1 and further ease the intensity of ferroptosis (38). The SLC7A11 gene codes for a sodium-independent cystine-glutamate anti-porter that is chloride dependent, known as system ${\text{X}}_{\text{c}}^{-}$ or xCT. As a component of the cysteine-glutamate transporter, SLC7A11 plays a central role in GSH homeostasis, which significantly protects cells from oxidative stress (39). GPX4 is a phospholipid hydroperoxidase that protects cells against membrane lipid peroxidation, and its activity can specifically inhibit ferroptosis (10). The increase of TFR1 mRNA abundance under a diquat challenge indicated that diquat induced mass Fe^3+^ into hepatocytes, which easily lead to hepatocellular lipid oxidation *via* the Fenton reaction. In addition, dietary HPE significantly increased hepatic HSPB1, SLC7A11, and GPX4 mRNA abundance in the diquat-challenged piglets. These results demonstrate that dietary HPE has a good effect on inhibiting ferroptosis through enhancing the hepatic antioxidant system, which is in agreement with the results of improved hepatic antioxidant capacity. In accordance with the results of mRNA abundance, the protein abundance results also showed that dietary HPE supplementation increased GPX4 and decreased TFR1 abundance, which demonstrates that dietary HPE is conducive to protect the liver from ferroptosis *via* inhibiting the transfer of Fe^3+^ and enhancing the synthesis of GPX4.

## Conclusion

In conclusion, HPE is a dietary supplement for relieving diquat-induced hepatic injury in the piglet model. HPE is able to relieve diquat-induced hepatic ferroptosis *via* inhibiting the transfer of Fe^3+^ and enhancing GPX4 expression.

## Data Availability Statement

All datasets generated in this study are included in the article/Supplementary Material.

## Ethics Statement

The animal study was reviewed and approved by the Animal Care and Use Committee of Wuhan Polytechnic University (Wuhan, China).

## Author Contributions

All authors were involved in study design and implementation, data acquisition, analysis, and interpretation. PH wrote the manuscript. HH, WT, HZ, YL, and XX read and approved the final version.

## Conflict of Interest

The authors declare that the research was conducted in the absence of any commercial or financial relationships that could be construed as a potential conflict of interest.

## Supplementary Material

Supplementary Figure 1 The original image for the blots of GPX4.

Supplementary Figure 2 The original image for the blots of SLC7A11.

Supplementary Figure 3 The original image for the blots of TFR1.

Supplementary Figure 4 The original image for the blots of β-actin.

## Abbreviations

ADP: adenosine diphosphate
ALT: alanine aminotransferase
AMP: adenosine monophosphate
AST: aspartate aminotransferase
ATP: adenosine triphosphate
BW: body weight
GPX4: glutathione peroxidase 4
GSH: glutathione
GSH-PX: glutathione peroxidases
GGT: glutamyl transpeptidase
HPE: holly polyphenols extracts
HPLC: high performance liquid chromatography
HSPB1: heat shock protein beta 1
MDA: malondialdehyde
ROS: reactive oxygen species
SLC7A11: solute carrier family 7 member 11
TAOC: total antioxidative capacity
TFR1: transferrin receptor protein 1.
