# Supplementary figures and images for: Holly (Ilex latifolia Thunb.) Polyphenols Extracts Alleviate Hepatic Damage by Regulating Ferroptosis Following Diquat Challenge in a Piglet Model
Source: Front Nutr. 2020 Dec 15;7:604328. doi: 10.3389/fnut.2020.604328 (PMC7770127; doi:10.3389/fnut.2020.604328)

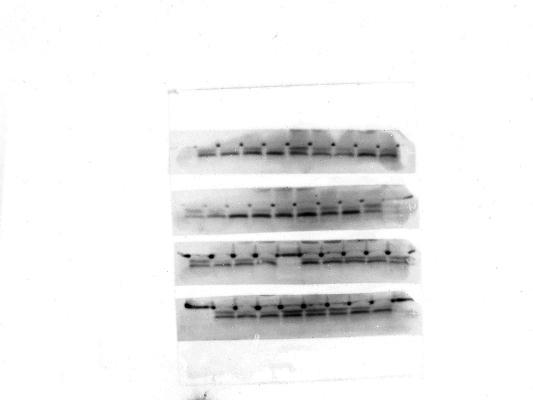

Supplement: Supplementary Figure 1 — The original image for the blots of GPX4. [file Image_1.JPEG]

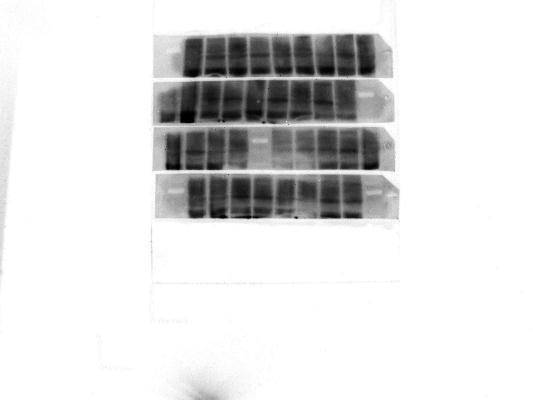

Supplement: Supplementary Figure 2 — The original image for the blots of SLC7A11. [file Image_2.JPEG]

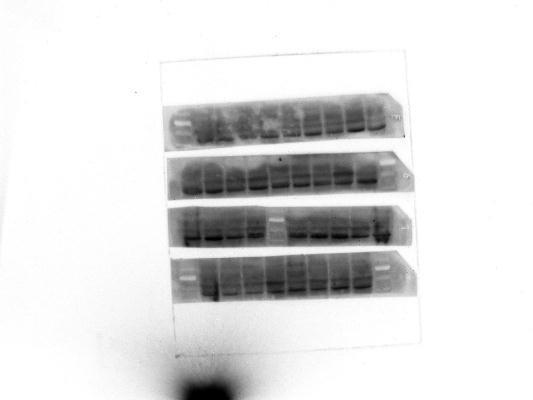

Supplement: Supplementary Figure 3 — The original image for the blots of TFR1. [file Image_3.JPEG]

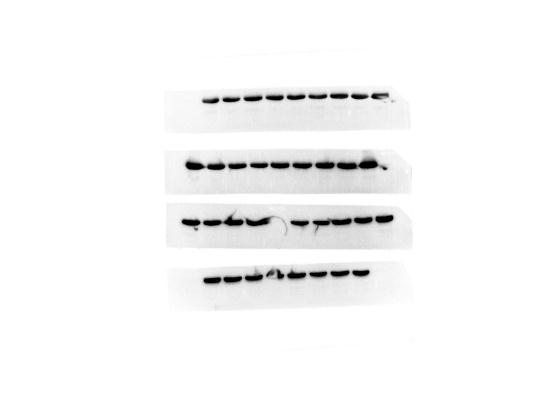

Supplement: Supplementary Figure 4 — The original image for the blots of β-actin. [file Image_4.JPEG]
